# Seasonal Variation and Impact of Waste-Water Lagoons as Larval Habitat on the Population Dynamics of *Culicoides sonorensis* (Diptera:Ceratpogonidae) at Two Dairy Farms in Northern California

**DOI:** 10.1371/journal.pone.0089633

**Published:** 2014-02-21

**Authors:** Christie E. Mayo, Cameron J. Osborne, Bradley A. Mullens, Alec C. Gerry, Ian A. Gardner, William K. Reisen, Christopher M. Barker, N. James MacLachlan

**Affiliations:** 1 Department of Pathology, Microbiology and Immunology, School of Veterinary Medicine, University of California Davis, Davis, California, United States of America; 2 Department of Health Management, Atlantic Veterinary College, Charlottetown, Prince Edward Island, Canada; 3 Department of Entomology, University of California Riverside, Riverside, California, United States of America; 4 Center for Vectorborne Diseases, University of California Davis, Davis, California, United States of America; Universidade Federal de Minas Gerais, Brazil

## Abstract

The Sacramento (northern Central) Valley of California (CA) has a hot Mediterranean climate and a diverse ecological landscape that is impacted extensively by human activities, which include the intensive farming of crops and livestock. Waste-water ponds, marshes, and irrigated fields associated with these agricultural activities provide abundant larval habitats for *C. sonorensis* midges, in addition to those sites that exist in the natural environment. Within this region, *C. sonorensis* is an important vector of bluetongue (BTV) and related viruses that adversely affect the international trade and movement of livestock, the economics of livestock production, and animal welfare. To characterize the seasonal dynamics of immature and adult *C. sonorensis* populations, abundance was monitored intensively on two dairy farms in the Sacramento Valley from August 2012– to July 2013. Adults were sampled every two weeks for 52 weeks by trapping (CDC style traps without light and baited with dry-ice) along N-S and E-W transects on each farm. One farm had large operational waste-water lagoons, whereas the lagoon on the other farm was drained and remained dry during the study. Spring emergence and seasonal abundance of adult *C. sonorensis* on both farms coincided with rising vernal temperature. Paradoxically, the abundance of midges on the farm without a functioning waste-water lagoon was increased as compared to abundance on the farm with a waste-water lagoon system, indicating that this infrastructure may not serve as the sole, or even the primary larval habitat. Adult midges disappeared from both farms from late November until May; however, low numbers of parous female midges were detected in traps set during daylight in the inter-seasonal winter period. This latter finding is especially critical as it provides a potential mechanism for the “overwintering” of BTV in temperate regions such as northern CA. Precise documentation of temporal changes in the annual abundance and dispersal of *Culicoides* midges is essential for the creation of models to predict BTV infection of livestock and to develop sound abatement strategies.

## Introduction

Members of the genus *Culicoides* are small (1–3 mm) hematophagous flies (midges) in the family Ceratopogonidae. They occur throughout the inhabited world, where they serve as biological vectors of several pathogenic animal viruses, including bluetongue virus (BTV), epizootic hemorrhagic disease virus, African horse sickness virus, and Akabane and Schmallenberg viruses [1], [2], [3], [4], [5], [6], [7], [8]. The diseases resulting from infection with these viruses impact international trade and movement of livestock, the economics of livestock production, and animal welfare [9], [10], [11], [12], [13]. Precise characterization of the seasonality of *Culicoides* midges is critical to the creation of accurate and ecologically sound parameter estimates for use in predictive models of the animal diseases transmitted by these midges and, ultimately, to the formulation of logical abatement strategies for their control [14], [15], [16], [17].

*C. sonorensis* is the primary vector of BTV among domestic and wild ruminants in the United States (U.S.) [3], [18], [19]. BTV infection is distinctly seasonal in temperate regions such as California (CA), where the vast majority of infections occur during the late summer and fall months [20], [21], [22], [23]. The virus largely disappears from ruminants resident in temperate regions from late fall/early winter until mid-summer (mid-November until late July in much of the Northern Hemisphere) [20], [24], [25], [26], [27]. Surveillance of sentinel livestock and serological and entomological surveys conducted on dairy farms within California have demonstrated high levels of BTV infection among both ruminants and vector midges during periods of peak virus transmission and vector activity (August – October) [22], [23], [28]. Dairy waste-water holding ponds (lagoons) are considered to be the primary developmental sites for *C. sonorensis* on intensive commercial dairy farms in California, because the immature stages develop in mud within the littoral zone of the ponds, which is heavily enriched with excrement [29], [30], [31], [32].

Since the recent European bluetongue epidemic, there has been a considerable emphasis on quantitatively defining aspects of the ecology of *Culicoides*, including adult emergence from immature habitats, habitat preference, and the dispersal of adults [33], [34], [35], [36], [37], [38], [39]. However, only a few published studies have utilized a transect study design to link information regarding the population dynamics of both immature and adult *C. sonorensis* on a local scale during the seasonal and non-seasonal periods of BTV infection of livestock to develop accurate parameter estimates to inform disease transmission models [40], [41], [42]. Therefore, the goals of the current study were to 1) compare the population dynamics of *C. sonorensis* on intensive commercial dairy farms with and without functioning waste-water lagoons during periods of the year with and without BTV infection of livestock in the Sacramento (northern Central) Valley of CA, 2) measure temporal changes in abundance and dispersion of adult midges on the same two study farms and 3) utilizing a transect study design, quantify the fine-scale effects of habitat type, biotic factors, and physiochemical conditions on the seasonal abundance and dispersion of *C. sonorensis*.

## Methods

Sampling of immature and adult *Culicoides* was conducted on two privately owned commercial dairy cattle farms (A and B) in Glenn County, CA (39.5900° N, 122.3900° W) for 52 weeks beginning August 16, 2012. The studies were conducted with the approval of the land-owners under strict confidentiality and did not involve endangered or protected species. This region was chosen because of its ecological diversity and data from prior surveillance that consistently demonstrated a high incidence of seasonal BTV infection among both cattle and *Culicoides* populations [23], [28]. Preliminary data confirmed that the maximal abundance of adult *C. sonorensis* occurred during late summer (July-September). The two farms were located 24 km apart at approximately the same latitude, and surveillance of *Culicoide*s has been ongoing at one of these farms for each of the three previous years (2009–2011). At Farm A, pastures are planted primarily with alfalfa and corn and are intersected by 1.5–2.0 m deep canals for drainage and irrigation. The dairy facility has concrete free stalls and dirt fields where most of the cattle are housed. Within the free stall area, waste was removed twice-daily from the concrete semi-slotted floors by a flush system. The slurry of waste was drained into the first of 5 waste-water lagoons, the contents of which were later used as fertilizer for the farm crops via an irrigation system. The largest waste-water lagoon, which served as a substantial breeding habitat for *C. sonorensis* throughout July, 2012 (data not shown) was drained, tilled, and the contents removed during the period of BTV transmission (August, 2012) allowing the evaluation of this intervention on the population dynamics of *C. sonorensis*. Other landscape features surrounding the farm included a forested area of native trees [Digger Pine (*Pinus sabiniana*); Valley Oak (*Quercus lobata*)] to the north and established black walnut orchards at varying distances to the north, south, and west. The second dairy farm (Farm B) was located 24 km to the West of Farm A and had similar dairy herd size, ecological features, and a stable waste-water lagoon infrastructure. There were no additional dairy waste-water lagoons or neighboring farms within 3 km of either farm (A or B).

### Trapping and collection of adult and immature *Culicoides sonorensis*

**Figure 1:**
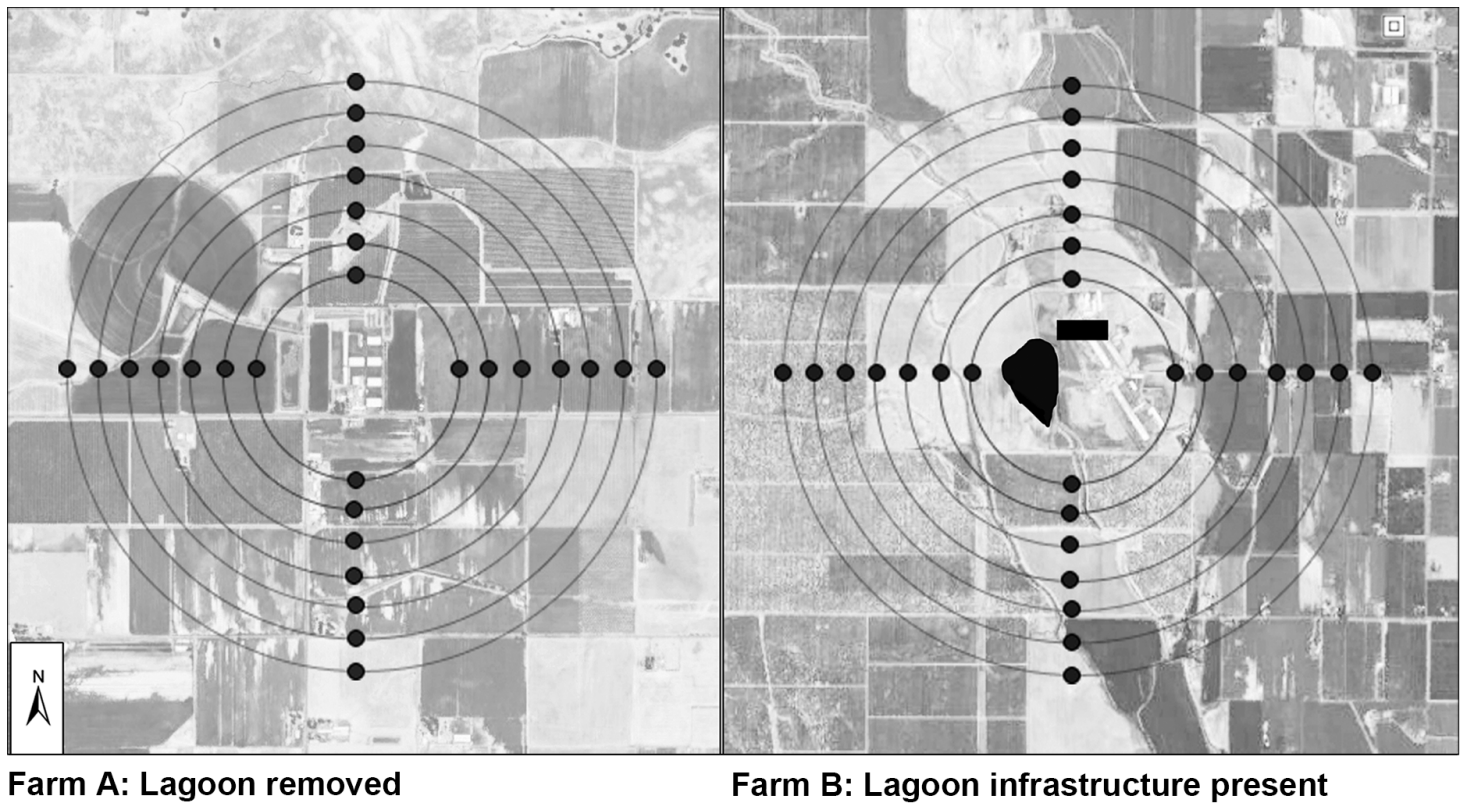
Map depicting 28 traps set in a transect array at 90° angles from the center of the waste-water lagoon at dairy farms where the lagoon infrastructure has been removed (Farm A) and retained (Farm B).

Trapping of adult *Culicoides* was performed every two weeks throughout the study period of 52 weeks (728 trap nights per farm) using CDC style traps without light and baited with dry-ice [43]. Traps were placed at 0.25 km intervals along 4 transects, each directed at 90° quadrants from the outside perimeter of the waste-water lagoons on Farm A and B, based on the assumption that the waste-water lagoons were the principal immature habitat of *C. sonorensis* (Figure 1). Traps were suspended 0.9 m above the ground and deployed 2 h before sunset and removed 2 h after sunrise. During the interseasonal BTV transmission period from late fall to early spring, temperatures were often too cold at sunset or sunrise for significant *Culicoides* flight activity; therefore, additional sampling was conducted from December 2012 to April 2013. Specifically, overnight collection bags were replaced with daytime collection bags each morning and the trap was allowed to run throughout the diurnal period. For optimal preservation and identification of anatomical structures, all insects were collected in deionized water containing 0.5% Alconox and sorted by sex and parity using standard stereomicroscopy. Parity was determined by the presence of pigment deposited in the abdominal cuticle [44] and by changes in the pigmentation pattern of the abdominal tergites [45]. While recent publications have indicated the need for development and validation of alternative and more sensitive methods in age grading adults, the method used in this study is the most widely used and easiest to employ on a large scale [37].

*Culicoides* midges were distinguished from other insects on the basis of their morphological characteristics and wing patterns using a stereomicroscope [46]. The *Culicoides variipennis (C. variipennis)* complex is separated into 3 discrete species: *C. sonorensis*, which is the primary vector of BTV in the western regions of North America and often associated with waste-water habitats; *C. occidentalis*, also a western North American species and typically localized to very saline or alkaline habitats; and, *C. variipennis*, which is present in eastern North America [47]. Females of *C. sonorensis* cannot be morphologically distinguished from *C. occidentalis*; therefore, the identity of female *C. sonorensis* was further supported by microscopic examination of a subset of slide-mounted male midges collected in the same traps from a random selection of traps in the transect.

Larvae were collected bi-weekly from the edge of the waste-water lagoon embankments on Farm B during early morning to limit desiccation as lagoon diameter varied with water level. Using a trowel, 30 ml samples of the surface mud (top 1–2 cm) were removed at 2.5 m intervals from along the edge of the lagoon, spanning the waterline (3–4 cm below to 3–4 cm above). Samples collected from the lagoon (∼20 per sample date) were pooled and returned in a wet ice cooler to the laboratory where they were warmed to room temperature, thoroughly homogenized, and a subsample of 30 ml immediately poured into a 150 ml beaker. Saturated MgSO4 (Epsom salts, 100 ml) was added to the mud, stirred, and the live larvae strained from the surface solution 3 min later [30]. Larvae of *C. sonorensis* were recognized under a dissecting microscope and enumerated by the four instars distinguished by head capsule size [30], [48].

### Physiochemical Conditions

For Farm B, lagoon water temperature and pH were recorded at 2 h intervals by a Datapod temperature logger (Datapod® CX6 series). Lagoon discharge and flow measurements were recorded daily utilizing a propeller meter. Information pertaining to management practices (waste management, lagoon flushing frequency, irrigation schedule, date of first crop irrigation, irrigation practices, crop cycles) were obtained from the managers of each of the two farms and confirmed by field observations. Monthly maximum and minimum temperature, precipitation, and vapor pressure values were extracted from the gridded meteorological surfaces for individual farms. Variables were obtained from weather (PRISM Climate Group, Oregon) and satellite imagery datasets (MODIS Land Products, ORNL DAAC). For all variables, weekly or monthly intervals were selected for aggregation to coincide with the surveillance of midge populations.

### Data analysis

Abundance of *C. sonorensis* on each farm was reported as adults per trap per night with midges enumerated by sex and parity status. Abundance of immature *C. sonorensis* was determined per instar from the waste-water lagoon on Farm B as previously described [30]. Data were checked for normality using the Kolmogorov-Smirnov test. Abundance data were not normally distributed, so a ln(y+1) transformation was used to approximate normality, control the variance and account for zero values during the non-seasonal period (December, 2012– April, 2013). Pearson linear correlations tested associations among weather parameters and the mean number of *Culicoides* collected per trap per sample day. Differences in mean numbers of adult *Culicoides* at each trap site location on individual farms were determined by a repeated measures analysis of variance (ANOVA). Means were separated by Tukey post-hoc multiple comparison tests. Parity rates were calculated as the number of parous females divided by the total female midges captured per trap night. Seasonal parity rates and mean midge abundance per trap were compared by repeated measures analysis of variance (ANOVA). For all statistical analyses, *p*<0.05 was considered significant. A local Moran I test for spatial autocorrelation was performed (SatScan 7.0 software, Kulldorf, M. and Information Mgmt. Services, 2004) as a second-order measurement for spatial dependency of *C. sonorensis* abundance on the trap sites of the uniform trapping grid.

## Results

### Temporal changes in populations of adult *C. sonorensis* on dairy farms with and without waste-water lagoons

**Figure 2:**
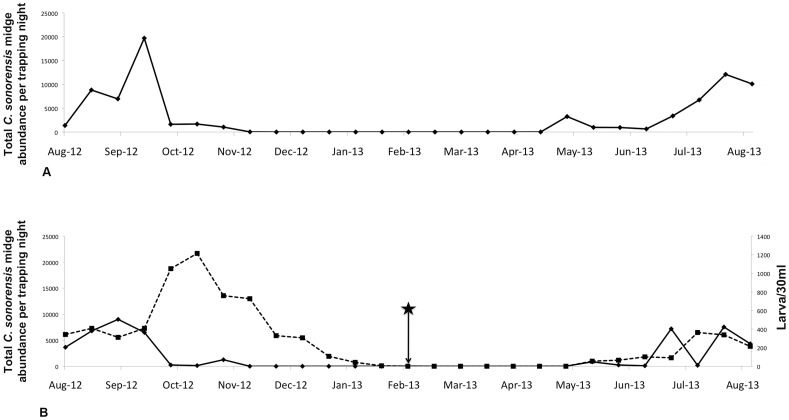
Seasonal abundance of *C.sonorensis* collected on each farm. A) Seasonal abundance of adult (adults per trap per day) *Culicoides sonorensis* collected on dairy Farm A; B) Seasonal abundance of immature (larvae/30 ml sample) (dashed line) and adult (adults per trap per day) (solid line) *Culicoides sonorensis* collected on dairy Farm B. The star denotes capture of 32 parous females during the month of February on the site with a lagoon present.

**Figure 3:**
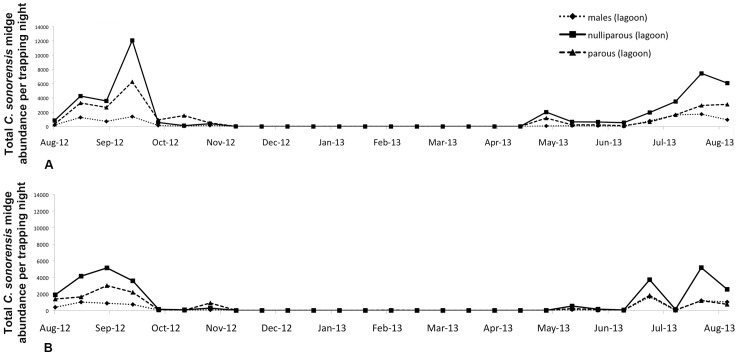
Seasonal abundance of adult *Culicoides sonorensis* distinguished by sex and parity (males- dotted line; parous-dashed line; nulliparous-solid line) for Farm A (top) and Farm B (bottom).

A total of 79,348 and 48,039 adult *C. sonorensis* (the only species trapped in this study) was collected from Farms A and B during 26 weeks of trapping, respectively; although not significant, increased numbers of adults per trap-night were collected from Farm A than Farm B despite the lack of a functioning waste-water lagoon at the former site. The peak collection of adults occurred in early September, 2012 on Farm A (mean, 13,327) and late September, 2012 on Farm B (mean, 7901) (Figure 2). With the highly notable exception of 32 parous females that were collected in daytime traps that were set on Farm B during February, 2013, *C. sonorensis* were not collected in traps set from late November, 2012 until May, 2013. The parameters significantly correlated with mean adult midge abundance on both farms during the period of peak activity (September, 2012) were mean minimum temperature (19°C, r = 0.34, p<0.05) and mean relative humidity (22.3%, 0.45, p<0.05). Parity rates were similar on both farms ranging from 0.18–0.45 on Farm A (mean, 0.35) and 0.19–0.67 on Farm B (mean, 0.38) (Figure 3). During the anticipated seasonal peak period of BTV transmission in September, 2012, nulliparous females were the most abundant portion of the total midge population on both farms; however, a parity shift occurred on both farms by late October, 2012, when parous females were in greater abundance than nulliparous females. The abundance of females was correlated with larval abundance on Farm B (r = 0.48, p<0.05), and time series correlations were improved by 2 week lag transformations (abundance of adults 1–2 weeks before first and second instars) (r = 0.62, p<0.05). Throughout the study, male midges were less abundant than female midges in traps operated on both farms, but the populations followed similar seasonal trends in abundance compared to the female midge population.

### Larval populations and physiochemical parameters of the waste-water lagoon on Farm B

**Figure 4:**
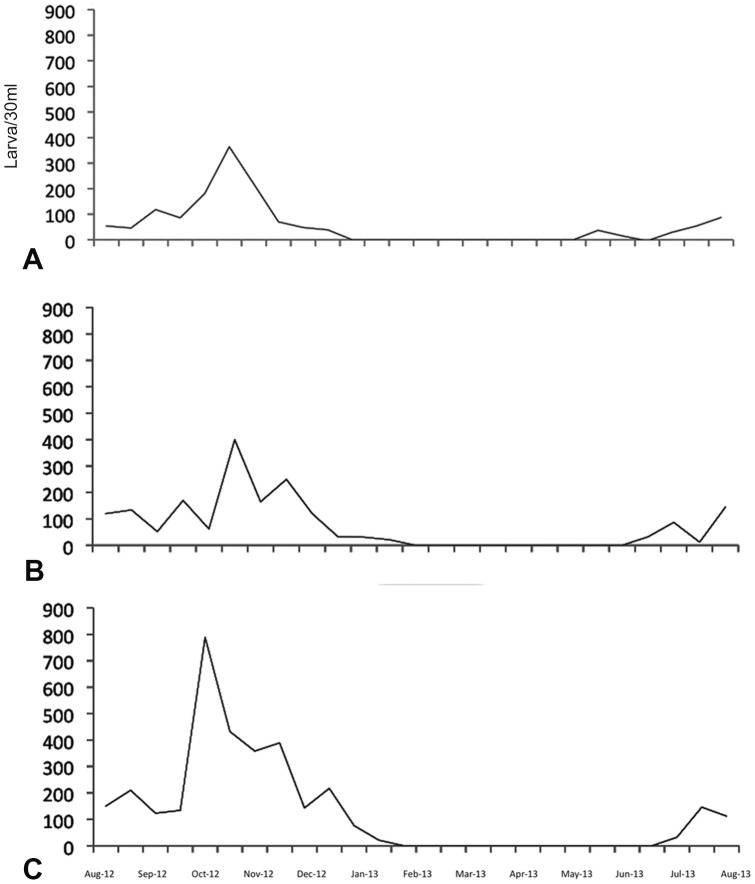
Seasonal *C. sonorensis* abundance for first and second (A), third (B), and fourth (C) instars.

Larval abundance at the waste-water lagoon on Farm B was greatest during the hot, dry period of the year (July-November) and lowest during the cold months (December-April) (Figure 4). Low numbers of early instars (L1 and L2) were recovered earlier in the season (May) than older L3 and L4 larvae (June-July); however, the peak abundance of early instars (late October) occurred after that of the later instars (early October) and adults (Figure 4). The mean temperature (26°C), pH (7.9) and effluent rate (2723.50 L/day) recorded from the waste-water lagoon on Farm B during the period preceding these larval peaks (June-August) were distinctly higher than those of previous months (November-May) (Table 1), and coincided with irrigation of the corn crop on the farm. During the months of peak larval abundance (August-October), the temperature was lower (22.3°C), lagoon water pH was higher (8.2), and corn was being harvested for production of silage on Farm B (Table 1). Although larval abundance was measured only on Farm B where a waste-water lagoon remained operational, the higher numbers of adult *C. sonorensis* throughout the season on Farm A (with no waste-water lagoon) stimulated us to seek additional larval habitat. Two irrigation culverts spanning approximately 0.75 km and located 0.25 km northwest of the evacuated lagoon on Farm A were identified as potential breeding sites and sampled. L3 and L4 larval instars were recovered on 3 sampling dates in August, 2013, confirming that these were suitable larval habitats.

**Table 1:** Mean seasonal physiochemical characteristics of waste water lagoon pond on dairy farm with lagoon infrastructure present and crop rotation/harvest schedule.

Physiochemical parameters	August-October, 2012	November, 2012- February, 2013	March-May, 2013	June-August, 2013
Temperature (°C)	22.3	13.7	15.9	26
Mean pH	8.2	7.5	7.6	7.9
Mean effluent flow rate (liters/day)	2275.14	1703.44	2850.42	2725.50
Crop rotation/harvest schedule	Cutting and harvesting of corn for silage	Planting of alfalfa	Harvest of alfalfa, planting corn	Maintenance and growth of corn

### Spatial associations among trap sites

**Figure 5:**
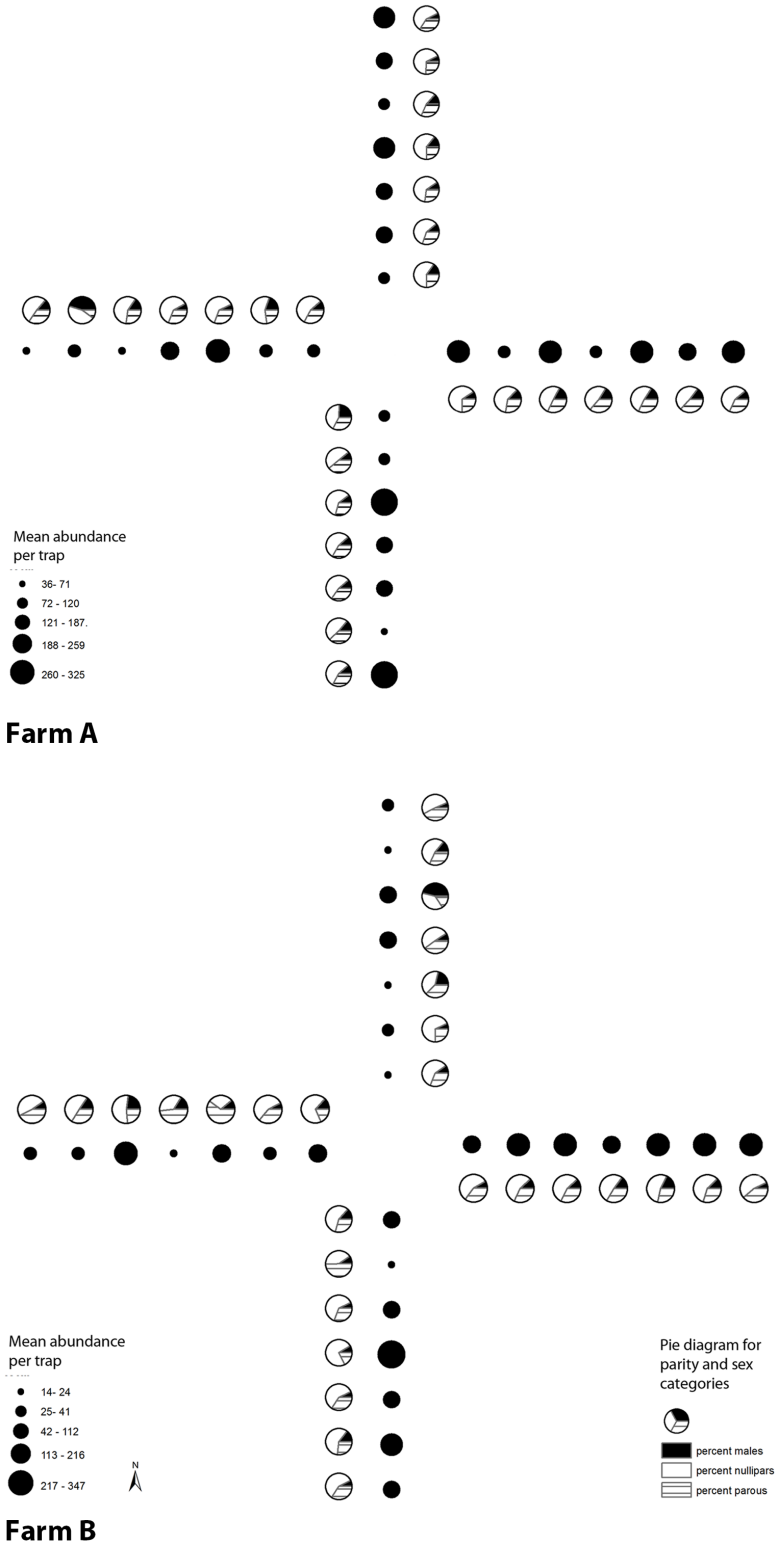
*Culicoides sonorensis* abundance (back-transformed geometric mean per trap day) at 28 trap sites along 4 transects (north, south, east, and west) on Farm A and Farm B. Abundance is represented by solid black circles, graduated in size. Further characterization of the proportions of males (black shading), nulliparous (white), and parous females (line shading are represented by pie diagrams for each trap.

Spatial associations among trap sites *C. sonorensis* abundance was heterogeneously distributed among traps on both farms and there was no significant difference in mean abundance among traps on either farm (Figure 5). The Moran I test for randomly distributed data showed no evidence of spatial clustering for *C. sonorensis* abundance (p>0.05) on Farm A or Farm B.

## Discussion

An intensive year-long entomological study provided several important results pertaining to the population dynamics of *Culicoides* populations on two intensive dairy farms in the Sacramento Valley of northern CA: (1) waste-water lagoons did not serve as the sole larval habitat on these farms as other potential habitats such as drainage culverts existed beyond the lagoon infrastructure, and draining of the lagoon on Farm A had no obvious significant impact on subsequent midge numbers on the farm as compared to those on a farm (B) with functioning lagoons; (2) parous female midges were active during daylight hours within winter, the interseasonal period of BTV transmission; (3) parous and nulliparous females were recovered in the spring during the period of greatest irrigation on the farms and before larval stages were recovered from the waste-water lagoon on Farm B; (4) nulliparous females predominated in collections made through the period of peak seasonal activity, a shift in overall parity occurred during transition to the interseasonal period; and (5) abundance of both adult and immature midges were correlated with specific and quantifiable physicochemical parameters throughout seasonal period of insect activity and BTV transmission.

Virtually all species of *Culicoides* require moist habitats for development of eggs, the four larval instars, and pupae [49], [50]. The association between development of immature *C. sonorensis* and manure-contaminated aquatic habitats, particularly dairy waste-water lagoons has been well-documented [31], [32], [49], [51], [52]. In the current study, however, the greater abundance of adult *C. sonorensis* on Farm A (lagoon removed) than Farm B, coupled with the identification of additional larval habitats (e.g. irrigation culverts) on Farm A and homogeneous dispersion of females among traps on Farm B, confirm that waste-water lagoons may not provide the only, perhaps not even the primary, larval habitat for *C. sonorensis* on intensive dairy farms and their environs in CA. Additionally, there could be differences in adult dispersion due to either females questing patterns or larval production sites that are not accounted for. In a statewide survey in 1983, 55% of dairy lagoons sampled supported early developmental stages of *C. sonorensis.* This finding does not, however, preclude the importance of other developmental sites, even as small as hoof print impressions [29], [53]. Therefore, although dairy waste-water lagoons clearly provide a suitable development site for *C. sonorensis*, the broad range of habitats that can potentially be exploited by *C. sonorensis* on CA dairy farms would suggest that: 1. identification and evaluation of all potential larval habitats is necessary to characterize the ecology of *C. sonorensis*; and 2. comprehensive larval control through lagoon habitat modification alone is unlikely to remove adults from agro ecosystems such as the Sacramento Valley where irrigated crops and riparian corridors are abundant.

With the very notable exception of the collection of 32 parous females on Farm B during February, 2013, continuous activity of adult *C. sonorensis* in the spring of 2013 occurred after a >5 month period when adults were not collected in traps, and prior to the detection of larvae in the waste-water lagoon on Farm B. Despite marked differences in ambient temperature, the beginning of stable seasonal activity of adult *C. sonorensis* in this study (early May, 2013) in northern CA was similar to that reported in southern Alberta (May), but considerably later than that reported in southern CA (February), Colorado (March), and New York (April) [30], [48], [54], [55]. Therefore, it is likely that the phenology of adult *C. sonorensis* may be influenced by both climatic and site-specific factors. For example, lagoon conditions (i.e. water level, edge slope, pollution) can change to impact their use by *C.sonorensis* over time [31]. Other potential explanations for the delayed emergence of adults in northern CA (as compared to other states of the U.S.) include the high proportion of third instars entering the non-seasonal period of midge activity in the fall and the late recovery of early instars in the spring, one week after the initial emergence of adults on Farm B.

Air and water temperatures should have been sufficient to allow at least some adult *C. sonorensis* activity on these dairies in spring, so the absence of detectable adult activity then is intriguing. The late recovery of early instars in spring, 1 week after adult activity began at Farm B, supports the idea that substantial vector activity simply did not occur earlier than that. Lagoon or irrigation ditch conditions (water level, edge slope, nutrient loading) can change very rapidly, and this would substantially impact their attractiveness for oviposition or development by immature *C. sonorensis*. In general, water levels are higher, and irrigation use much less, in cooler winter and spring periods. The delayed activity of adults in northern California therefore also might reflect the relative lack of irrigation on these dairies in winter and spring, and the lack of immature habitats that irrigation might create for *C. sonorensis*. Higher water levels in lagoons or ditches also often result in steeper edge slopes, and more edge vegetation, both of which tend to disfavor larval *C. sonorensis* abundance and development.

The collection of 32 parous females during Feb 2013 when mean temperatures ranged from 5–15°C shows unequivocally that parous adult female *C. sonorensis* were active during the winter period. This is supported by other studies where adult daytime activity of *C. sonorensis* has been documented during the Spring and Fall throughout Colorado and California [48], [56]. There are several potential, non-exclusive explanations: 1. older females oviposited in the fall and then survived until they became active and were collected in February; 2. Blood-fed female midges entered quiescence in the fall and remained gravid until warm temperature stimulated oviposition and then host-seeking behavior during winter; or 3. larval stages remained viable throughout the winter and emerged as host seeking females. However, if the latter were the true would be anticipated that nulliparous females should have been collected, unless they emerged earlier and we collected only the older remnants of this cohort. Although our study was focused on the seasonal dynamics of *C. sonorensis*, the collection of diurnally active parous females host-seeking in mid-winter (February) provides important insight into a potential “overwintering” mechanism for BTV within temperate regions. This finding is also consistent with the sporadic isolations of BTV from cattle in CA as early as April and prior to the reputed transmission period [21]. Furthermore, it was shown previously that *C. variipennis* (later elevated to *C. sonorensis*) can survive for up to 53 days at cold temperatures in the laboratory [57], and others have shown that survival of adult *C. sonorensis* is inversely related to temperature [55], [58]. Thus, it is quite possible that BTV might over-winter in northern California through a low-level cycle of infection between susceptible livestock and long-lived parous females, which remain persistently infected with BTV for their entire lifespan. This mechanism was proposed by Nevill (1971) for the over-wintering of BTV in temperate regions of South Africa, and the possibility has been considered in Europe based on adult *Culicoides* collected in the winter [25], [59], [60].

Abundance of adult and immature *C. sonorensis* increased as mean temperature increased throughout the season. The seasonal trend and rate of change in midge abundance was similar on both farms, and likely reflected temperature-dependent development of immature stages and subsequent emergence of adults [58]. Warm temperatures were associated with reduced levels of parity and increased populations of nulliparous females, whereas cooler temperatures were associated with a shift to increased parity late in the season. These findings are consistent with those of earlier studies that showed a late season increase in parity [61], [62]. This could reflect reduced recruitment of nulliparous females, in combination with increased survival of females at lower temperatures as seen in previous studies [55]. In addition to temperature, the seasonal patterns of abundance of immature and adult midges were correlated with waste-water lagoon pH and other specific lagoon effluent values on Farm B. The effluent measurements are surrogate indices for irrigation practices, as dairy waste-water lagoons are often drained to fertilize and maintain crops (primarily corn) throughout the spring and summer months. It is not surprising that greater abundance of both immature and adult *C. sonorensis* were associated with increased effluent flow, as previous studies on CA dairy farms have shown that the drainage of lagoons with gently sloping banks provided a moist habitat for larval development and increased populations of *Culicoides* [23], [30], [32], [53]. The importance of temperature in determining midge abundance was consistent with the increased rate of development of immature stages and subsequent emergence of adult midges as temperature increases [58]. The significance of an association between *C. sonorensis* populations and higher water pH is uncertain but consistent with prior studies that have documented *C. sonorensis* to be more tolerant than other midge species to fresh, salt, or alkaline water habitats, although few data are available pertaining to the advantage, if any, for larval development [29], [49], [63].

The results obtained in the current investigation contrast somewhat from those of prior entomological surveys undertaken on intensive CA dairy farms in other regions of the state, and identify several potential ecological and seasonal influences on populations of immature and adult *Culicoides* that have been previously ignored. The data are especially relevant to the better characterization of the complex cycle of *Culicoides* transmitted virus infections of livestock such as BTV and, in particular, potential mechanisms of inter-seasonal maintenance (“overwintering”) of these agents. Importantly, our studies provided key objective and quantifiable parameter estimates, specifically seasonal activity and dispersion of *C. sonorensis* on California dairy farms, for the creation of ecological models to guide the formulation of logical abatement and disease mitigation strategies.
