# Correction: Seasonal Variation and Impact of Waste-Water Lagoons as Larval Habitat on the Population Dynamics of *Culicoides sonorensis* (Diptera:Ceratpogonidae) at Two Dairy Farms in Northern California

**DOI:** 10.1371/journal.pone.0107336

**Published:** 2014-09-02

**Authors:** The PLOS ONE Staff

The following information is missing from the Funding section: CM Barker receives additional support from the Research and Policy for Infectious Disease Dynamics (RAPIDD) program of the Science and Technology Directorate, Department of Homeland Security and Fogarty International Center, National Institutes of Health.
